# Elastographic Histogram Analysis as a Non-Invasive Tool for Detecting Early Intestinal Remodeling in Experimental IBD

**DOI:** 10.3390/jcm14113992

**Published:** 2025-06-05

**Authors:** Rareș Crăciun, Marcel Tanțău, Cristian Tefas

**Affiliations:** 1 Department of Internal Medicine, ”Iuliu Hațieganu” University of Medicine and Pharmacy, 400008 Cluj-Napoca, Romania; craciun.rares.calin@elearn.umflcluj.ro (R.C.); tefascristian@gmail.com (C.T.); 2 Gastroenterology Clinic, ”Prof. Dr. O. Fodor” Regional Institute of Gastroenterology and Hepatology, 400162 Cluj-Napoca, Romania

**Keywords:** elastography, intestinal fibrosis, inflammation, strictures, TNBS colitis, intestinal fibrosis, inflammatory bowel disease, strain imaging, non-invasive diagnostics, ulcerative colitis, animal model

## Abstract

**Background/Objectives**: Inflammatory bowel disease (IBD), encompassing Crohn’s disease and ulcerative colitis, is characterized by cycles of inflammation and tissue remodeling that can culminate in fibrosis. Differentiating between early inflammatory and fibrotic bowel wall changes remains a diagnostic challenge due to overlapping imaging features. This study aimed to assess the potential of elastography, specifically pixel histogram analysis, as a non-invasive method to identify acute inflammatory changes in a rat model of 2,4,6-trinitrobenzenesulfonic (TNBS)-induced colitis. **Methods**: Female CRL:Wi rats were randomized into control and experimental groups, with the latter receiving intracolonic TNBS to induce acute colitis. On day 7 post-induction, all animals underwent ultrasonographic and strain elastographic assessment of the distal colon using a standardized protocol. Histogram-based analysis of red, green, and blue pixel distributions was performed on elastographic video frames. Results were compared with histologic grading of inflammation and fibrosis using hematoxylin-eosin and Masson’s trichrome staining. **Results**: Rats with TNBS-induced colitis exhibited significant weight loss, increased bowel wall thickness (31.5% vs. controls, *p* < 0.01), and elevated elastographic pixel intensity across all color channels (*p* < 0.05). Histologically, experimental animals showed severe inflammation and early submucosal fibrosis. A strong positive correlation was found between elastographic histogram values and histologic fibrosis scores (r = 0.86, *p* < 0.01), confirming the technique’s diagnostic relevance. **Conclusions**: Elastographic pixel histogram analysis is a reproducible, non-invasive approach capable of distinguishing acute inflammatory changes and early fibrotic remodeling in experimental colitis. These findings support its potential application as a diagnostic adjunct in the early assessment and monitoring of IBD-related bowel wall changes.

## 1. Introduction

Inflammatory bowel diseases (IBD) are chronic, relapsing-remitting disorders of the gastrointestinal tract that significantly impact patients’ quality of life [1,2]. Crohn’s disease (CD) and ulcerative colitis (UC), the two main forms of IBD, are characterized by episodes of acute inflammation that can lead to progressive structural damage [3]. While long-term complications such as fibrosis and stricturing are well-documented, the acute inflammatory response plays a pivotal role in disease progression and symptom severity. Repeated cycles of inflammation and healing trigger a cascade of biological events that contribute to changes in bowel wall architecture, including increased vascular permeability, tissue edema, and early extracellular matrix deposition. These modifications alter the mechanical properties of the intestinal wall and can have significant implications for disease management [4,5].

A major challenge in clinical practice is distinguishing between active inflammation and the initial stages of fibrosis, as both processes contribute to bowel wall thickening and altered tissue mechanics. While chronic inflammation leads to excessive extracellular matrix deposition and scarring, even acute inflammatory episodes can induce significant tissue remodeling [6]. Edema, increased collagen turnover, and transient fibrotic activity can result in increased tissue stiffness, mimicking chronic fibrotic changes. Differentiating these alterations is essential for selecting the most appropriate therapeutic strategy, albeit remaining a major challenge in clinical practice [6]. Patients with predominantly inflammatory strictures may respond well to intensified medical therapy, including corticosteroids and biologic agents, whereas those with significant fibrotic involvement often require endoscopic or surgical intervention [7,8].

Current imaging modalities such as computed tomography (CT), magnetic resonance imaging (MRI), and contrast-enhanced ultrasonography (CEUS) provide valuable insights into bowel wall alterations but lack the precision needed to directly assess early fibrotic changes [9]. Histological evaluation remains the gold standard for identifying fibrosis, but it is invasive and not feasible for routine clinical monitoring. Thus, there is a critical need for non-invasive tools capable of reliably distinguishing between inflammatory and early fibrotic changes in IBD.

Acute inflammation in IBD is associated with significant bowel wall thickening due to edema and inflammatory infiltration, both of which can alter tissue elasticity. Given its ability to assess mechanical properties of the intestinal wall in real time, elastography could serve as a valuable tool for evaluating these early inflammatory changes. Unlike conventional imaging techniques, elastography offers real-time, quantitative measurements of tissue elasticity, making it a practical tool for longitudinal disease monitoring and treatment assessment. However, its clinical utility in differentiating acute inflammation from early fibrotic remodeling still requires full validation [10,11,12,13,14].

In this study, we aimed to investigate the early inflammatory changes in the intestinal wall induced by TNBS colitis in a rat model and to assess the potential of elastography to differentiate between normal and inflamed tissue. The TNBS-induced colitis model is widely used to mimic key aspects of human IBD, particularly the initial response seen in CD. By focusing on this early disease phase, we sought to determine whether elastography could reliably detect bowel wall alterations associated with acute inflammation. If successful, elastography could become a valuable addition to the clinical armamentarium, offering a reliable, non-invasive method for assessing disease activity and guiding therapeutic strategies in IBD.

## 2. Materials and Methods

### 2.1. Animals and Housing

Female CRL:Wi rats aged between 8 and 10 weeks were used in the present study. The animals were housed in standard open-top cages and had access to filtered tap water and pelleted feed ad libitum. Standard aseptic wood chip bedding was used. The rats were bred and maintained in the Laboratory Animal Facility of the “Iuliu Hațieganu” University of Medicine and Pharmacy, Cluj-Napoca, Romania, at a controlled temperature of 22 °C ± 2 °C and relative humidity of 55% ± 10%, under a 12:12 h light/dark cycle (lights on, 7 am to 7 pm) with a light intensity of 300 lx at 1 m above the floor. The animals were randomly allocated into two groups: experimental (TNBS-induced colitis) group and control group. Rats were individually marked at the base of the tail using permanent markers. All procedures were approved by the Ethics Committee of the “Iuliu Hațieganu” University of Medicine and Pharmacy and the Romanian Competent Authority in accordance with EU Directive 2010/63. Before starting the experiment, all animals underwent a 7-day acclimatization period. Environmental enrichment was provided using autoclaved braided cotton dental rolls and wooden sticks. The in vivo study was designed and performed following the ARRIVE Guidelines for Reporting Animal Research [15].

### 2.2. Induction of Colitis and Experimental Procedures

Colitis was induced in the experimental group rats using 2,4,6-trinitrobenzenesulfonic acid (TNBS). General anesthesia was achieved by intraperitoneal administration of 80 mg/kg Ketamine and 8 mg/kg Xylazine. TNBS was dissolved in 50% ethanol in a 1:1 ratio and administered intracolonically using a 6 Fr medical catheter inserted transanally and advanced 8 cm. Each rat received 0.8 mL of the TNBS + ethanol solution. To achieve uniform administration of TNBS intracolonically, the catheter was gently retracted while the syringe content was slowly instilled into the colon. After administration, the rats were maintained in a vertical position for 5 min to ensure uniform distribution of the solution throughout the colon. The control group received 0.8 mL of 50% ethanol in saline solution using the same procedure. The instillation was repeated daily for three consecutive days. During this period, the animals were maintained on a liquid diet in the morning and afternoon.

On day 7 after the initial administration, ultrasonographic and elastographic evaluations were performed on all animals. Following imaging, rats were euthanized by cervical dislocation under general anesthesia, and the entire colon from anus to ileocecal junction was harvested for histological examination.

### 2.3. Examination Protocol

Ultrasonography and elastography were conducted using an Ultrasonix probe (CBT Biotechnologies, Toronto, Canada) with a 6.5 MHz linear probe. The same brightness, contrast, intensity, and gain settings were used for all ultrasound examinations. Since numerical elastography data was displayed using a rainbow-coded scale with values ranging from 1 to 256, modifications in system settings did not affect further analysis. All examinations were performed by two physicians with different levels of experience. The probe was positioned in a convenient location in the hypogastric region. Three 10 s video recordings were taken by each examiner for each subject, with the region of interest set to include the sigmoid colon. The videos were stored uncompressed at maximum quality for precise analysis.

### 2.4. Ultrasonographic Examination

Following ultrasonographic localization of the rat colon, the wall thickness was measured (mm). Five values were obtained for each rat, and their mean value was used as a reference.

### 2.5. Elastographic Examination

**Figure 1:**
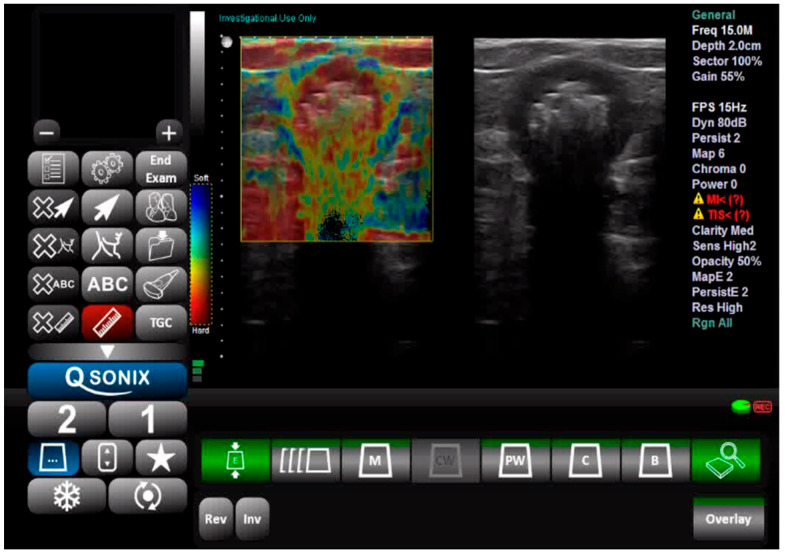
Ultrasound and elastography assessment of the sigmoid colon.

**Figure 2:**
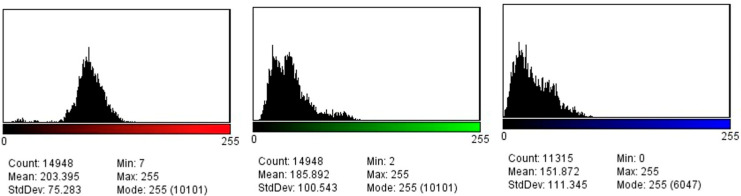
Red, green, and blue pixel histograms generated after the elastography assessment.

Tissue elasticity was reconstructed within the region of interest and translated into a color-coded signal superimposed on the grayscale image. To visualize tissue elasticity, different elasticity values corresponded to different colors (ranging from 1 to 256). The system was set to use a red/green/blue color map, where hard tissue areas were marked in red, intermediate tissues in green, and soft tissue areas in blue (Figure 1). The full spectrum from blue to red was applied to each elastographic determination. The degree of tissue compression was evaluated using a numerical scale from 1 to 5 in each image. Each recorded elastographic video was subjected to dynamic computer analysis using image processing software (ImageJ, version 1.53i, NIH, University of Wisconsin, Milwaukee, WI, USA). Histograms were generated for the three primary colors (red/green/blue) from a representative frame for each video, with each hue corresponding to an intensity value based on the number of pixels occupied by that hue in the selected frame (Figure 2). The numerical values thus obtained were subsequently analyzed statistically.

Due to equipment limitations, the elastographic assessment was based on histogram color distribution rather than absolute stiffness values in kilopascals (kPa). The analysis focused on comparing the relative intensity of red, green, and blue components between experimental and control groups, without predefined stiffness cutoffs. The RGB pixel distribution was used as a surrogate marker for tissue stiffness based on the color-encoding schema of the elastography software, where red represents stiffer regions and blue indicates softer tissue. Similar approaches using color histogram analysis have been validated in other fields, such as thyroid elastography and pancreatic lesions. This method offers a semi-quantitative and reproducible means of assessing tissue mechanical properties in the absence of absolute kPa values [16,17].

To ensure consistency, imaging was performed in the distal colon, with particular attention to including the sigmoid and rectal regions in the field of view. A representative frame was selected from each elastographic video by identifying the moment with maximal probe-to-surface contact and optimal elastographic signal homogeneity, as agreed upon by both examiners. To ensure reproducibility, the same selection criteria were applied across all subjects and recordings. Inter-observer variability in frame selection was minimized by joint review of candidate frames prior to histogram extraction.

### 2.6. Histological Evaluation

The colons were excised in their entirety, from the anus to the ileocecal junction, opened longitudinally, and cleaned. Macroscopic examination showed that the TNBS-treated colons were indurated, edematous, and thickened, with evident ulceration and mucosal bleeding. Tissue samples from affected regions were then collected and fixed in formalin. Serial paraffin-embedded sections of the colon were stained with hematoxylin and eosin to evaluate inflammation severity and with Masson’s trichrome to assess fibrosis. The grading of colitis and fibrosis was undertaken based on previously developed scores available in the literature (Table 1 and Table 2) [18,19].

### 2.7. Statistical Analysis

A certified biomedical statistician was used for the statistical analysis utilizing SPSS 29.0.1.0 software (SPSS Inc., Chicago, IL, USA). Continuous variables with normal distribution were compared using Student’s *t*-test, and results were considered statistically significant at a *p*-value below 0.05. The control group was compared with the experimental group for all three histogram variants (red, green, blue), wall thickness, colitis histology score, and fibrosis score.

## 3. Results

A total of 40 female Crl:WI rats, aged between 8 and 10 weeks and weighing 257.5 ± 58.5 g were included in the study. These rats were selected for their age and weight range to ensure uniformity and representativeness of the experimental cohort. A total of 20 were allotted to the experimental group and 20 to the control group. The introduction of TNBS led to significant weight loss from an average of 255.2 g on day 0 to 238.05 g on day 7 (*p* < 0.01), corresponding to a 6.72% decrease in body weight, and development of liquid bloody stools in all exposed animals. One rat from the control group and two rats from the experimental group died during the first five days.

Regarding the ultrasonically measured thickness of the anterior intestinal wall, we observed a significant difference between the mean values, with the control group averaging 1.11 mm and the experimental group averaging 1.46 mm (*p* < 0.01), a 31.5% increase in size (Table 3).

The elastographic evaluation revealed significantly higher mean stiffness values in TNBS-induced colitis tissues compared to the control group (*p* = 0.005 for blue histograms; *p* = 0.01 for green histograms and *p* = 0.02 for red histograms) (Table 3). The inter-observer agreement was high, with a reproducibility index of 0.89, indicating reliable consistency in elastographic measurements. Since no predefined stiffness thresholds were established, elastographic changes were assessed by comparing mean histogram values between groups rather than applying absolute differentiation criteria. This approach allowed for a direct evaluation of relative tissue stiffness alterations in TNBS-induced colitis, highlighting significant differences in color distribution between inflamed and control tissues.

Histologically, TNBS-treated rats showed considerable mucosal inflammation, necrosis, and less epithelial regeneration (Table 3). Submucosal fibrosis was notably more pronounced in TNBS-induced tissues, with a strong correlation observed between histological fibrosis grading and elastographic stiffness (r = 0.86, *p* < 0.01) (Table 3).

## 4. Discussion

Our study demonstrates that pixel histogram-based elastographic analysis can effectively differentiate early inflammatory changes in TNBS-induced colitis, revealing a promising non-invasive approach for assessing bowel wall alterations in IBD. By employing a TNBS-induced colitis model, we documented significant increases in bowel wall thickness and tissue stiffness, with strong correlations between histologic inflammation, fibrosis scores, and elastographic histogram values. This is particularly beneficial in differentiating transient inflammation-induced stiffness from early fibrotic changes—a clinical challenge currently unmet by most imaging modalities and can significantly alter therapeutic decisions [9].

The prior literature has extensively explored the role of elastography in chronic IBD. An Italian study group demonstrated that ultrasound elasticity imaging could predict ileal fibrosis in CD with high accuracy (AUC of 0.917) when combined with conventional sonographic signs [10]. However, the study protocol compared patients with CD scheduled for the surgical treatment of fibrotic strictures and a control group of patients with CD in remission, thus delineating between two opposites (certain fibrosis vs. no inflammation). Similarly, Chen et al. applied real-time shear wave elastography in a clinical setting to differentiate fibrotic from inflammatory strictures in Crohn’s disease patients, showing significantly higher stiffness values in fibrotic tissues [14]. Our findings build upon this by applying elastographic evaluation to the initial remodeling phase, a time window where differentiation is notoriously difficult using standard imaging. Notably, the histogram-based technique allowed for a quantifiable and semi-automated evaluation of the data, circumventing common subjectivity and inter-operator variability often observed with conventional qualitative elastography [11,12].

Studies, such as those by Stidham et al. and Kim et al. have validated elastography in both animal models and human subjects, primarily focusing on differentiating chronic fibrosis from inflammation [12,13]. These studies, while instrumental in establishing the method’s clinical relevance, predominantly emphasize later-stage disease, often missing early inflammation that may still be reversible. Our work fills this gap by capturing subtle increases in submucosal stiffness and early fibrosis markers, offering a potential tool for earlier therapy and avoiding unnecessary surgical management [7,20].

In particular, our histogram analysis of the recordings echoes previous findings that used shear wave velocity measurements in TNBS models to separate inflamed from fibrotic bowel segments [11,21]. While their studies relied on more advanced shear wave systems, our method, based on color histogram analysis of strain elastography, offers a cost-effective and easily deployable alternative suitable for preclinical and potentially clinical settings lacking high-end modules. Moreover, the observed pixel distribution shifts in our study correspond well with histologic stages of inflammation and fibrotic remodeling, validating histogram analysis as a surrogate for biomechanical assessment.

The TNBS-induced model remains one of the most widely accepted experimental platforms for studying acute colitis due to its reproducibility and resemblance to Crohn’s-like inflammation, including transmural infiltration, crypt distortion, and ulceration [18,19]. Despite its limitations in modeling chronicity and the full spectrum of fibrosis seen in human IBD, this model is ideal for testing elastographic sensitivity in early disease phases. Our findings mirrored histologic severity, especially in rats with submucosal fibrotic involvement, indicating that pixel-based elastography could serve as a proxy for identifying histopathological progression. This aspect is particularly valuable considering the ethical and logistical constraints of performing serial biopsies in human IBD patients [6].

From a pathophysiological standpoint, the increase in bowel wall thickness and stiffness in acute TNBS colitis is driven mainly by mucosal and submucosal edema, inflammatory cell infiltration, and increased extracellular matrix (ECM) turnover [4,5]. These factors increase tissue turgor and viscoelasticity, which elastography is designed to detect. Importantly, our data suggest that even without full-thickness fibrosis, the histogram shifts can indicate early ECM remodeling, reinforcing that inflammation and fibrosis exist on a continuum [3,6]. This supports previous findings where fibrosis correlated with increasing tissue stiffness but lacked sensitivity in early changes due to insufficient resolution of standard imaging [10,14].

A particularly novel element of our study is quantifying data via histogram pixel analysis. While not a direct measure of stiffness in kPa like shear wave techniques, our method allows relative comparison of tissue mechanical properties and enhances reproducibility through semi-automated analysis. Given the inter-operator variability that plagues conventional elastography [12], our reproducibility index of 0.89 suggests high intra-observer consistency and feasibility for broader application. Similar histogram approaches have been used in oncology and liver fibrosis assessment, but their use in bowel disease, especially IBD, remains underexplored [16,17].

Nonetheless, our study is not without limitations. First, the reliance on strain elastography and pixel distribution lacks the absolute quantification capability of shear wave elastography, which might limit standardization across different platforms. Second, the use of an acute TNBS model does not fully simulate the chronic fibrotic environment seen in longstanding IBD, thereby limiting generalizability. Third, while we correlated elastographic results with histology, the analysis was confined to a short-term, seven-day inflammation window. Longitudinal studies tracking the progression from acute inflammation to chronic fibrosis are necessary to validate histogram-based elastography as a biomarker for disease trajectory. Additionally, our study’s reliance on RGB histogram analysis, though objective, may be influenced by minor variations in acquisition settings despite efforts to standardize image parameters.

Our findings also raise important translational implications. In clinical practice, differentiating between inflammatory and fibrotic strictures remains a major diagnostic challenge. Current imaging modalities, including MRI and CT enterography, provide anatomic information but lack sufficient resolution for early fibrosis detection [9]. Contrast-enhanced ultrasound adds functional data but is similarly limited in mechanical characterization. Real-time elastography, particularly with standardized histogram-based analysis, could fill this diagnostic void, offering bedside assessment of tissue elasticity and facilitating tailored treatment decisions. This is especially relevant in the context of emerging biologics and small molecules where treatment responsiveness depends on underlying tissue composition. Standardization of image acquisition, color calibration, and software-based histogram extraction would be required for clinical implementation. Integration with real-time elastographic platforms and prospective validation in IBD patients would be the next essential steps and might require industry collaborations for real-time artificial intelligence-assisted analysis. If the method is further validated and gains traction, establishing normative and pathological RGB thresholds could aid in the diagnostic classification of inflammatory versus fibrotic strictures and become a reliable decision-making tool for treating structuring Crohn’s disease.

## 5. Conclusions

In summary, our study supports the integration of elastographic histogram analysis into IBD diagnostics, particularly in preclinical models. By demonstrating significant correlations between elastographic color distribution and histological markers of inflammation and fibrosis, we provide a proof-of-concept that non-invasive, pixel-based elastographic assessment can differentiate early inflammatory remodeling from normal tissue. This approach addresses a notable gap in current imaging modalities and offers a scalable tool for both research and potential clinical applications.

## Figures and Tables

**Table 1:** Histological grading of colitis.

Feature	Score	Description
Inflammation	0	None
	1	Mild
	2	Moderate
	3	Severe
Damage/necrosis	0	None
	1	Mild
	2	Moderate (involving muscularis mucosa)
	3	Severe (transmural, involving muscularis propria)
Regeneration	0	Complete re-epithelization
	1	Broad, multifocal re-epithelization
	2	Focal, migration, and mitotic features
	3	None

**Table 2:** Histological grading of fibrosis.

Score	Description
0	No fibrosis
1	Fibrosis in the submucosa
2	Fibrosis in the mucosa and submucosa
3	Fibrosis in the muscularis mucosa, submucosa, and mucosa
4	Fibrosis in muscularis propria, muscularis mucosa, submucosa, and mucosa
5	Full thickness fibrosis, including serosa

**Table 3:** Experimental data.

	Control Group	Experimental Group	*p*-Value	Effect Size (Cohen’s D)
Weight (grams)				
Day 0	256.6 (199–316)	255.2 (194–334)		
Day 7	249.89 (196–313)	238.05 (180–328)		
Histogram average value (range)				
Red	165.94 (160.81–172.45)	172.56 (154.62–205.38)	0.02	0.72
Green	144.44 (122.09–168.08)	156,28 (137.82–187.42)	0.01	0.80
Blue	136.22 (112.12–159.11)	149.96 (131.27–185.22)	*p* < 0.01	0.91
Wall thickness				
Day 0	1.08 (0.99–1.15)	1.1 (1.02–1.2)	*p* < 0.01	
Day 7	1.11 (1.02–1.19)	1.46 (1.19–1.72)	*p* < 0.01	
Colitis histology score	6.63 (4–9)	1.84 (0–3)	*p* < 0.01	
Fibrosis score	0.5 (0–1)	1.67 (1–3)	*p* < 0.01	

## Data Availability

The raw data supporting the conclusions of this article will be made available by the authors on request, given that the dataset is part of an ongoing research project.

## Abbreviations

The following abbreviations are used in this manuscript:

IBD	Inflammatory bowel disease
CD	Crohn’s Disease
UC	Ulcerative colitis
MRI	Magnetic Resonance Imaging
CT	Computed Tomography
CEUS	Contrast-enhanced ultrasonography
TNBS	2,4,6-trinitrobenzenesulfonic acid
